# A Streamlined Approach to Prader-Willi and Angelman Syndrome Molecular Diagnostics

**DOI:** 10.3389/fgene.2021.608889

**Published:** 2021-05-11

**Authors:** Samuel P. Strom, Waheeda A. Hossain, Melina Grigorian, Mickey Li, Joseph Fierro, William Scaringe, Hai-Yun Yen, Mirandy Teguh, Joanna Liu, Harry Gao, Merlin G. Butler

**Affiliations:** ^1^Fulgent Genetics, Temple City, CA, United States; ^2^Department of Psychiatry and Behavioral Sciences and Pediatrics, University of Kansas Medical Center, Kansas City, KS, United States

**Keywords:** streamlined molecular diagnostics, whole-exome sequencing, copy number variants, point mutations, methylation status, Prader–Willi syndrome, Angelman syndrome

## Abstract

Establishing or ruling out a molecular diagnosis of Prader–Willi or Angelman syndrome (PWS/AS) presents unique challenges due to the variety of different genetic alterations that can lead to these conditions. Point mutations, copy number changes, uniparental isodisomy (i-UPD) 15 of two subclasses (segmental or total isodisomy), uniparental heterodisomy (h-UPD), and defects in the chromosome 15 imprinting center can all cause PWS/AS. Here, we outline a combined approach using whole-exome sequencing (WES) and DNA methylation data with methylation-sensitive multiplex ligation-dependent probe amplification (MLPA) to establish both the disease diagnosis and the mechanism of disease with high sensitivity using current standard of care technology and improved efficiency compared to serial methods. The authors encourage the use of this approach in the clinical setting to confirm and establish the diagnosis and genetic defect which may account for the secondary genetic conditions that may be seen in those with isodisomy 15, impacting surveillance and counseling with more accurate recurrence risks. Other similarly affected individuals due to other gene disorders or cytogenetic anomalies such as Rett syndrome or microdeletions would also be identified with this streamlined approach.

## Introduction

**FIGURE 1:**
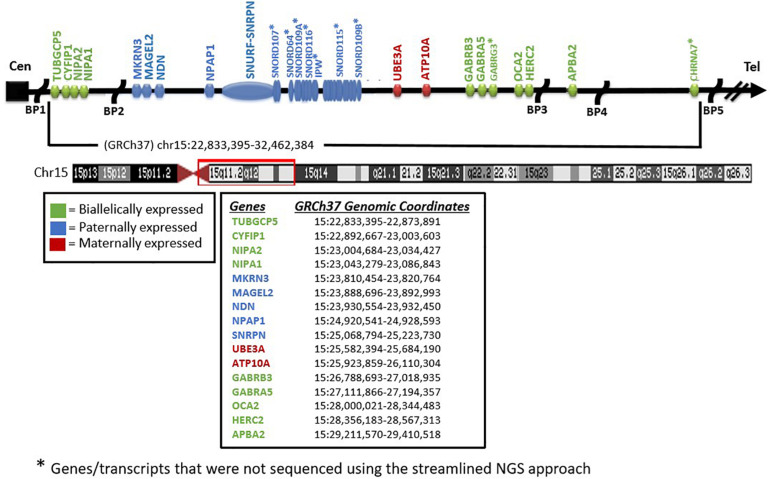
Chromosome 15 ideogram. Genes and previously reported breakpoints (“BP”) in the 15q11-q13 region are shown in their relative genomic positions. Genes not marked with *asterisk* were sequenced by the whole-exome sequencing test performed.

As reviewed previously, testing for Prader–Willi and Angelman syndromes (PWS/AS) has historically required a stepwise approach taking up valuable time and resources (e.g., Velinov et al., 2000; Martínez et al., 2006; Ramsden et al., 2010; Poole et al., 2013; Beygo et al., 2019; Butler et al., 2019a; Butler and Duis, 2020). By applying multiple analytical methodologies to whole-exome sequencing (WES) data (Hartin et al., 2019) for the identification of copy number changes and combined with methylation-sensitive multiplex ligation-dependent probe amplification (MLPA) (e.g., Henkhaus et al., 2012), one can deduce both the diagnostic status and most likely molecular mechanism in a single clinical report. Three different analytical modules are required for the WES analysis: sequence variant analysis including both the *SNRPN* and *UBE3A* genes, copy number analysis of the same and neighboring genes within the 15q11-q13 region, and absence of heterozygosity (AOH) analysis on chromosome 15q (see Figure 1). Sequence variant analysis from WES has been described on multiple occasions in the literature, while copy number analysis is more challenging. To address this challenge, the identification of the copy number variants (CNV) of three or more consecutive exons is utilized as an in-house developed method based on the comparison of normalized coverage to batch controls generating very high sensitivity. However, the limited specificity of this method is ameliorated by the use of methylation-sensitive MLPA, which includes copy number and methylation-specific probes for analysis. AOH can be identified by analyzing the zygosity status of the common variants typically filtered out with the WES analysis. The distribution and density of these variants can vary across the genome and individuals, but segmental uniparental isodisomy (i-UPD; > 5 million bases) or total isodisomy of the entire chromosome 15q arm can be detected with high sensitivity and specificity. Microdeletions of the imprinting center can also be detected in PWS (Tan et al., 2020).

The largest PWS cohort analyzed to date and reported by Butler et al. (2019a) showed that 61% of patients with PWS have the typical 15q11-q13 deletion, either the larger type I or smaller type II involving chromosome 15q11-q13 proximal BP1 or distal BP3 breakpoints in type I or proximal BP2 and distal BP3 breakpoints in type II. The second most common genetic finding is maternal disomy 15 (uniparental disomy, UPD) seen in 35% of PWS patients in which both 15 s are inherited from the mother and grouped into three subclasses (maternal heterodisomy 15, segmental isodisomy 15, and total isodisomy 15). The remaining patients have imprinting defects (microdeletions or epimutations) or other chromosome 15 abnormalities including translocations.

## Subjects and Methods

### Patient Selection and Specimens

To test the accuracy of the streamlined molecular genetic testing approach outlined for PWS/AS, a series of 28 individuals (12 males and 16 females; average age, 37 ± 10 years) with an established clinical and molecular diagnosis of Prader–Willi syndrome with 15q11-q13 deletion subtypes, maternal disomy 15 subclasses, and imprinting defects was collected from a clinical genetics practice at the University of Kansas Medical Center directed by one of the coauthors (MGB). Of these 28 PWS subjects, the molecular genetic class, sex, and age were deidentified and assigned a case number prior to submitting the samples to Fulgent Genetics for use with the streamlined approach under study. Four of the subjects had the typical larger 15q11-q13 type I deletion, five had the typical smaller 15q11-q13 type II deletion, five had maternal segmental isodisomy 15, five had maternal heterodisomy 15, five had maternal total isodisomy 15, two had imprinting center defects due to a microdeletion, and two had non-deletion (epimutation) imprinting center status. The molecular genetic class information was not shared with the laboratory until the conclusion of the study. Prior to DNA isolation for genetic testing, the patients and/or guardians reviewed and signed consent forms for research approved by the local IRB for research on human subjects.

**FIGURE 2:**
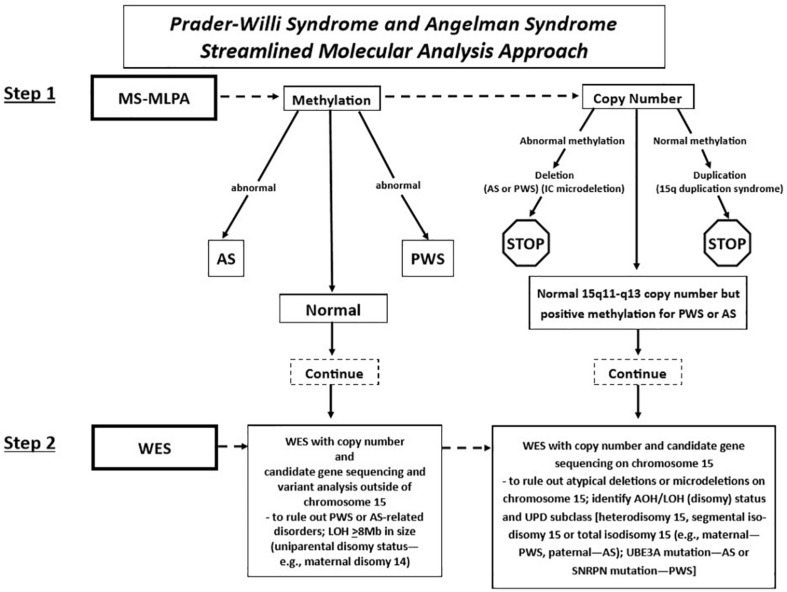
Prader–Willi syndrome and Angelman syndrome molecular analysis workflow. The approach begins with methylation-sensitive MLPA (MS-MLPA) to determine the methylation status and copy number of the 15q11-q13 region (step 1). Based on the results of step 1, proceed to step 2, with whole-exome sequencing (WES) as illustrated in the flowchart for the determination of copy number status, sequencing of genes on chromosome 15 and elsewhere with the determination of AOH and/or LOH. *UPD*, uniparental disomy; *PWS*, Prader–Willi syndrome; *AS*, Angelman syndrome; *AOH*, absence of heterozygosity; *IC*, chromosome 15q11 imprinting center; *LOH*, loss of heterozygosity.

Fulgent Genetics, a CLIA-approved commercial laboratory for genetic testing, undertook the streamlined approach and generated a molecular genetic report on each case and submitted the test results to the clinician (coauthor MGB submitting the DNA samples) with molecular genetic findings [(i.e., DNA methylation status, deletion subtype, UPD subclass, and imprinting center defect finding: microdeletion vs. non-deletion (epimutation)] on each subject (Figure 2).

As reference and supporting evidence of genetic testing experience for Fulgent Genetics (Temple City, California, United States), patients submitted for testing from January 1, 2018 through July 31, 2020 were used anonymously without specific identifying information.

For copy number and AOH analyses, a series of 297 randomly selected control individuals was selected from a pool of clinical laboratory results. All controls had available data on the testing platform used for the patient samples, and none of these individuals had a clinical diagnosis of PWS/AS. Normal control patient samples or cell lines were used for all MLPA assays.

### Laboratory Methodology

#### Methylation-Specific Multiple Ligation-Dependent Probe Amplification

Methylation-specific MLPA was performed for all patients using the SALSA MLPA Probemix ME028 Prader–Willi/Angelman kit (MRC Holland, Amsterdam, the Netherlands) following protocols published previously (e.g., Henkhaus et al., 2012) by one of the coauthors (MGB). MS-MLPA is a variation of the multiplex PCR method allowing the amplification of multiple targets with a single primer PCR pair, thereby detecting copy number changes at the molecular level of genes within the 15q11-q13 region as well as outside. The methylation status can be determined based on the methylation properties of the imprinted genes (e.g., *SNRPN*) within the 15q11-q13 region for both PWS and AS (see Figure 1).

#### Whole-Exome Sequencing

Whole-exome sequencing was performed using clinically validated methods (e.g., College of American Pathologists, Northfield, IL, United States). Briefly, DNA was extracted from whole blood specimens using standard methods. Next-generation sequencing (NGS) library preparation was performed using the KAPA HyperPlus Kit [reference no. 07962428001, Roche Holding AG (“Roche”), Basel, Switzerland]. Target capture was performed routinely using a custom probe mix based on IDT xGen Exome Research Panel v2 (Integrated DNA Technologies Inc., Coralville, IA, United States). Customization includes additional genes and intervals as well as rebalancing of probe amounts to maximize coverage and uniformity. Sequencing was performed routinely using the NovaSeq 6000 System (Illumina Inc., San Diego, CA, United States). Next-generation sequencing libraries were generated using modified versions of the KAPA DNA Library Preparation Kits (Roche Sequencing, Pleasanton, CA). This library preparation method used enzyme cocktails to fragment chromosomal DNA, perform end repair, and ligate adapters. The fragmented DNA was then amplified by standard PCR protocols, which simultaneously added sample-specific barcodes. Once amplified, the fragmented genome was selected for regions of interest using a hybrid of proprietary in-house and commercial capture set probes (Integrated DNA Technologies Inc., Coralville, IA). The selected regions of interest were large enough so that the selection did not need to be customized on a test-by-test basis. After an enrichment PCR protocol, sequencing by synthesis was performed on an Illumina HiSeqX or NovaSeq6000 instrument (Illumina, Inc., San Diego, CA) (see Figure 2).

### Bioinformatics

#### Small Variants

Sequence alignment and variant calling were performed as routinely done in the commercial laboratory setting using Sentieon’s germline variant calling pipelineDNAseq (v2018.08.05) with the reads aligned to a modified version of the hs37d5 reference (Sentieon Inc., San Jose, CA, United States) (Kendig et al., 2019). All genomic regions in this article use human genome reference Feb. 2009 (GRCh37/hg19). Raw data are available *via* the NCBI Short Read Archive (SRA) at https://www.ncbi.nlm.nih.gov/bioproject/687521.

#### Gene Variant Pathogenicity Criteria

Genetic variants were classified using technical standards established by the American College of Medical Genetics and Genomics (ACMG), Association for Molecular Pathology (AMP), and ClinGen (Richards et al., 2008, 2015; Riggs et al., 2020).

### Absence of Heterozygosity

A custom algorithm called AOHdetector was developed and routinely used in the laboratory setting and available for this study on PWS and AS testing. The tool is similar in approach to others such as PLINK (Purcell et al., 2007) or GERMLINE (Gusev et al., 2009). The AOHdetector parses a VCF (Variant Call Format) file, categorizes each variant listed as homozygous, heterozygous, or ignored (within a segmental duplication region), and identifies chromosome intervals over which there are homozygous variants and no heterozygous variants (Pei et al., 2020). The categorized variants are grouped by chromosome and then sorted by nucleotide position. Intervals are formed by finding neighboring, non-ignored variants that are of the same zygosity. The ignored variants have no effect on how the intervals are formed; however, they are tabulated for reference. Intervals are not allowed to span into or across a centromere for a chromosome. Based on internal data and expectations based on AOH [also called “runs of homozygosity” (ROH) or “autozygosity”] patterns in published studies, blocks of AOH larger than 1.5 Mb are relatively rare, whereas smaller blocks are very common (Ceballos et al., 2018; Clark et al., 2019). Specifically, for the 15q11 locus, assessment of patterns across 297 control individuals with internally available exome data revealed that all control individuals had at least 16 heterozygous variants within a 5-Mb region surrounding the *SNRPN* locus (boundaries used GRCh37; chr15:22,892,936–27,892,936). To test the efficacy of the AOHdetector in the context of PWS/AS testing, the number of heterozygous variant calls within a 5-Mb critical region on chromosome 15 (e.g., chr15:22,892,936–27,892,936) that include both *SNRPN* and *UBE3A* along with other genes within the 15q11-q13 region between breakpoints BP1 and BP3 were made from the 297 controls. No AOH blocks were detected in these controls. The average number of heterozygous variants in this region across this set was 42.5 (*SD* = 12.76). This establishes that > 99% of normal individuals will have four or more heterozygous single nucleotide variants (SNVs) in this interval. Individuals with four or fewer would then be suspected of having the absence of heterozygosity, which can be caused by a deletion of one allele or i-UPD. Specimens were considered to be positive for the 15q11 AOH region if they had fewer than four heterozygous SNVs in the interval, while loss of heterozygosity (LOH) was defined as greater than 5 Mb in size and if the patient has a methylation signal consistent with either PWS or AS. If the methylation status is unknown, then the size should be 8 Mb or greater to be considered an LOH designating uniparental disomy 15 and not present in other chromosomes to rule out consanguinity (Papenhausen et al., 2011; Butler et al., 2019a). The X chromosome is not analyzed for males with a 46,XY karyotype.

### Copy Number Analysis

Potential CNV were called using an internal coverage-based tool called CNVexon. This is a laboratory-developed tool similar to other methods such as ExomeCNV (PubMed: 21828086) and ExomeDepth (PubMed: 28378820). The coverage of each target exon is normalized against batch controls co-sequenced using the same captureset. A minimum of four comparators is required. Typical analyses contain more specimens (up to 48 for whole exome). Exons with a coverage ratio outside ABS(1 - N) = 0.2 (e.g., < 0.8 for deletions and > 1.2 for duplications) are marked as potential CNV, and a confidence score is given as a score equal to the ratio of the difference between the observed internally normalized coverage for the specimen and the mean normalized coverage across the comparators divided the standard deviation of the normalized coverage across the comparators. This score is thus a doubly normalized (intra-specimen and inter-specimen) coverage *Z* score with the number of standard deviations above/below the mean. Contiguous exons flagged in the same direction are then grouped with their *Z* scores considered independent. Groups with a combined score of ≥ 5 SD from the mean with at least one exon having a ratio ≤ 0.6 or ≥ 1.4 are considered potentially positive. Copy number analysis of *SNRPN*, *UBE3A*, and additional neighboring genes (e.g., *NIPA1*, *NIPA2*, *CYFIP1*, *TUBGCP5*, *MAGEL2*, *MKRN3*, *NDN*, *NPAP1*, *ATP10A*, *GABRA5*, *GABRG3*, *OCA2*, *HERC2*, and *APBA2*) was included to enable delineation of the typical larger 15q11-q13 type I deletion having *NIPA1* and *NIPA2* genes deleted and located between the 15q11.2 BP1–BP2 region or the typical smaller 15q11-q13 type II deletion where these two genes would be intact (Burnside et al., 2011; Butler, 2016; Rafi and Butler, 2020; see Figure 2). These typical deletions are seen in both PWS and AS. Also, atypical 15q11-q13 deletions that are larger or smaller than the typical deletions are seen in about 7% of patients with PWS or AS as a cause (Beygo et al., 2019; Butler et al., 2019a; Butler and Duis, 2020) and would be identified with this streamlined approach for molecular diagnostics. The results were compared with the MLPA copy number assessment for orthogonal confirmation.

This method is capable of reliably detecting deletions/duplications of three consecutive exons with > 99% sensitivity. Deletions/duplications of two consecutive exons are detected with > 98% sensitivity. Single-exon del/dup sensitivity is estimated at 96%. These sensitivity estimates are based on a retrospective analysis of 10,587 quantitative PCR (qPCR) reactions performed across 2,047 specimens at 8,543 unique genomic loci. As the specificity of this NGS-based CNV deletion/duplication calling is low (positive predictive values of 45 and 34% for deletions and duplications, respectively), confirmatory testing using an orthogonal method such as MLPA or qPCR is required. Calls with ≥ 12 consecutive exons deleted/duplicated are an exception, with > 99% positive predictive value, and thus represent an exception where confirmatory testing may not be necessary.

### Methylation Analysis

Methylation-specific MLPA utilizes 47 CNV probes within and/or outside of the 15q11-q13 region and five separate probes for the analysis of individual methylation status encompassing two separate imprinted genes (*SNRPN* and *MAGEL2*) for the identification of methylation patterns in comparison with the non-imprinted genes in this region. For example, the typical methylation intensity signals from patients with PWS are located usually between 80 and 100, while the intensity signals seen in control individuals range from 40 to 60 (Butler, 2011, 2016; Henkhaus et al., 2012).

## Results

### Head-to-Head Analysis

Blinded concordant results were achieved for all 28 of 28 specimens (Tables 1, 2). Nine individuals were expected and observed to have the type I or type II deletions involving 15q11-q13 breakpoints BP1 and BP3 or BP2 and BP3, respectively, by NGS copy number and confirmed by MLPA copy number analysis.

**TABLE 1:** Expected and observed results for 28 test patients with Prader–Willi syndrome.

Patient no.	Gender	Overlapping AOH (> 2 Mb)	Copy number variant (CNV)	Methylation status (MLPA)	Expected result	Streamlined result
1962659	F	Deletion overlap	Large deletion	Loss of paternal allele	Type II deletion, paternal	Same as expected
1962652	F	Deletion overlap	Large deletion	Loss of paternal allele	Type II deletion, paternal	Same as expected
1962648	M	Deletion overlap	Large deletion	Loss of paternal allele	Type I deletion, paternal	Same as expected
1962655	F	Deletion overlap	Large deletion	Loss of paternal allele	Type I deletion, paternal	Same as expected
1962651	F	Segmental isodisomy 15, i-UPD	Negative	Loss of paternal allele	Segmental maternal isodisomy 15, i-UPD	Same as expected
1962656	M	Deletion overlap	Large deletion	Loss of paternal allele	Type II deletion, paternal	Same as expected
1962644	M	Deletion overlap	Large deletion	Loss of paternal allele	Type I deletion, paternal	Same as expected
1962650	F	Deletion overlap	Large deletion	Loss of paternal allele	Type II deletion, paternal	Same as expected
1962672	M	Deletion overlap	*SNRPN* deletion	Loss of paternal allele	ICD (deletion of *SNRPN*)	Same as expected
1962636	F	Segmental isodisomy 15, i-UPD	Negative	Loss of paternal allele	Segmental maternal isodisomy 15, i-UPD	Same as expected
1962665	F	Total isodisomy15, i-UPD	CNV fail	Loss of paternal allele	Total maternal isodisomy 15, i-UPD	Same as expected
1962661	M	Total isodisomy15, i-UPD	Negative	Loss of paternal allele	Total maternal isodisomy 15, i-UPD	Same as expected
1962660	M	Segmental isodisomy 15, i-UPD	Negative	Loss of paternal allele	Segmental maternal isodisomy 15, i-UPD	Same as expected
1962649	M	Segmental isodisomy 15, i-UPD	Negative	Loss of paternal allele	Segmental maternal isodisomy 15, i-UPD	Same as expected
1962667	F	None	CNV fail	Loss of paternal allele	Maternal heterodisomy 15	M-het-UPD or ICD (unknown)
1962662	F	Total isodisomy15, i-UPD	Negative	Loss of paternal allele	Total maternal isodisomy 15, i-UPD	Same as expected
1962670	M	None	CNV fail	Loss of paternal allele	Maternal heterodisomy 15	M-het-UPD or ICD (unknown)
1962669	F	None	Negative	Loss of paternal allele	Maternal heterodisomy 15	M-het-UPD or ICD (epimutation)
1962657	F	Segmental isodisomy 15, i-UPD	Negative	Loss of paternal allele	Segmental maternal isodisomy 15, i-UPD	Same as expected
1962675	F	None	Negative	Loss of paternal allele	ICD (copy neutral), chromosome 15 biparental inheritance	M-het-UPD or ICD (epimutation)
1962668	F	None	Negative	Loss of paternal allele	Maternal heterodisomy 15	M-het-UPD or ICD (epimutation)
1962666	M	None	Negative	Loss of paternal allele	Maternal heterodisomy 15	M-het-UPD or ICD (epimutation)
1962671	M	None	Negative	Loss of paternal allele	ICD (copy neutral), chromosome 15 biparental inheritance	M-het-UPD or ICD (epimutation)
1962664	F	Total isodisomy15, i-UPD	CNV fail	Loss of paternal allele	Total maternal isodisomy 15, i-UPD	Same as expected
1962663	M	Total isodisomy15, i-UPD	CNV fail	Loss of paternal allele	Total maternal isodisomy 15, i-UPD	Same as expected
1962673	F	None	*SNRPN* deletion	Loss of paternal allele	ICD (deletion of *SNRPN*)	Same as expected
1962654	F	Deletion overlap	Large deletion	Loss of paternal allele	Type I deletion, paternal	Same as expected
1962639	M	Deletion overlap	Large deletion	Loss of paternal allele	Type II deletion, paternal	Same as expected

**AOH, absence of heterozygosity; CNV, copy number variant analysis; ICD, imprinting center defect; i-UPD, uniparental isodisomy; M-het-UPD, maternal uniparental heterodisomy.**

**TABLE 2:** Known molecular mechanisms of PWS and AS and the expected results for the different analytical methods employed.

Genetic analysis/methodology	Scenario I: multi-gene copy number change	Scenario II: single gene copy number change	Scenario III: point mutation	Scenario IV: i-UPD	Scenario V: h-UPD	Scenario VI: imprinting center defect
WES variant analysis	Negative	Negative	*SNRPN*: PWS *UBE3A*: AS Other WES identified genes (e.g., *MAGEL2*); Rett or other related disorders	Negative	Negative	Negative
WES copy number analysis	Multi-gene del/dup	Single/partial gene del/dup	Negative	Negative	Negative	Negative^a^
AOH/LOH	Dependent on size of deletion	Negative	Negative	Positive	Negative	Negative
MS-MLPA	Abnormal pattern	Abnormal pattern	Normal pattern	Abnormal pattern	Abnormal pattern	Abnormal pattern
MLPA copy number	Deletion	Deletion	Negative	Negative	Negative	Negative^a^

**WES, whole-exome sequencing using next-generation sequencing; i-UPD, uniparental isodisomy (two subclasses: segmental and total isodisomy); h-UPD, uniparental heterodisomy; PWS, Prader–Willi syndrome; AS, Angelman syndrome; AOH, absence of heterozygosity; LOH, loss of heterozygosity; MS, methylation sensitive; MLPA, multiplex ligation probe amplification analysis of 15q11.2 genes.^*a*^SNRPN or imprinting center microdeletion can be detected.**

Nine individuals were expected and observed to have maternal i-UPD by NGS AOH analysis and methylation-sensitive MLPA (loss of methylation at the paternal *SNRPN* and *MAGEL2* loci in PWS). These cases represent a mixture of total chromosomal 15 isodisomy and segmental isodisomy 15. The AOH algorithm applied was able to identify which samples were due to segmental isodisomy 15 with the number and size of segments including their location and those with total isodisomy 15 due to errors in maternal meiosis I and meiosis II, respectively, this is important for genetic counseling and surveillance for other at-risk genetic conditions, particularly if the mother (in PWS) or the father (in AS) is a carrier of pathogenic autosomal-recessive gene variants on chromosome 15 in patients with these UPD subclasses (Driscoll et al., 1998; Bittel et al., 2006; Gold et al., 2018; Manzardo et al., 2018; Beygo et al., 2019; Butler et al., 2019a,b; Butler and Duis, 2020).

Seven individuals were expected and observed to have maternal uniparental heterodisomy (h-UPD) by the detection of normal copy number, normal number of heterozygous variants in the locus, and methylation-sensitive MLPA (loss of methylation at the paternal *SNRPN* and *MAGEL2* loci in PWS). Those with maternal heterodisomy or h-UPD lack crossover events in maternal meiosis I and are not at risk of having a second genetic condition related to recessive gene mutations on chromosome 15. As WES with next-generation sequencing is undertaken, one can scan hundreds of autosomal-recessive genes on chromosome 15 for pathogenic variants in the isodisomic regions that could account for additional genetic conditions due to the presence of two identical alleles. In addition, females with PWS may be at risk of having X-linked disorders due to skewed X chromosome inactivation from the trisomy 15 rescue event occurring during early embryonic development (e.g., Cassidy et al., 1992; Butler et al., 2007). This may lead to a single cell in the trisomic rescued embryo having the potential for all subsequent cells with the same active X chromosome with extreme X chromosome inactivation skewness. The same X chromosome which is now active in the developing female embryo with PWS and UPD may lead to the expression of a pathogenic variant and the presence of an X-linked condition, as seen in affected males without PWS (Butler et al., 2007; Butler, 2016). There are hundreds of genes on the X chromosome by which females with PWS having any UPD subclass may be at risk of developing X-linked disorders requiring surveillance.

Two individuals were expected to have imprinting center defects. In both cases, methylation-sensitive MLPA (loss of methylation at the paternal *SNRPN* locus) was consistent with this finding. Defects of *SNRPN* were only detected by NGS and confirmed by MLPA, indicating the presence of these imprinting center defects as microdeletions in the imprinting center region. One of these deletions based on NGS analysis spans at minimum chr15:25,200,019–25,223,890 (see Figure 1). This region is 23 kb and may not be detectable by certain microarray platforms depending on the probe density and laboratory settings. The two remaining patients had non-deletion status of the imprinting center, indicating epimutation status confirmed by undertaking genotyping of polymorphic chromosome 15 markers using DNA from the PWS child and parents not undertaken in this streamlined approach. About 4% of all patients with genetically confirmed PWS by DNA methylation studies will have imprinting center defects, and about 20% of those will have microdeletions of the imprinting center detected with this streamlined approach (Hartin et al., 2018, 2019; Butler et al., 2019a). Historically, the remaining PWS patients (approximately 3% of all PWS patients) are those without segmental or total isodisomy 15 status or have typical or atypical 15q11-q13 deletions. Microdeletions of the imprinting center are identified using high-resolution chromosomal microarrays. Additional testing such as genotyping of chromosome 15 markers using parental DNA would determine whether biparental (normal) inheritance of chromosome 15 markers is present in the PWS child and therefore is due to epigenetic (non-deletion) status and not from non-deletion maternal heterodisomy 15 status (Butler et al., 2019a; Butler and Duis, 2020). The streamlined molecular diagnostic approach we describe will identify microdeletions as well as point mutations in the imprinting center and therefore should eliminate the need for additional testing using genotyped chromosome 15 markers with parental DNA.

In our study, copy number analysis could not be performed by NGS alone for five cases submitted for analysis [marked “Not tested (CNV fail)” in Table 1]. NGS-based CNV analysis is sensitive to DNA quality, and these were archival specimens not extracted with this analysis in mind. The typical CNV fail rate for DNA extracted from whole blood specimens is <1% (internal lab data, Fulgent Genetics). None of the 297 control individuals had a deletion or AOH signal consistent with a potential PWS/AS diagnosis, indicating high specificity for this approach.

### Additional Cases and Genes

When collated and analyzed, more than 6,100 individuals using hereditary genetic panels (e.g., panels containing “Intellectual Disability” or “Autism” from January 2018 to July 2020), no pathogenic small variants were reported in the *SNRPN* gene, indicating the uniqueness of this gene showing genetic variation. Deletions involving the *SNRPN* gene were found in six individuals (four with clinical signs and symptoms consistent with AS and two with PWS). Both point mutations (10 individuals) and whole/partial gene deletions (five individuals) were found in the *UBE3A* gene. Of note is that *UBE3A* was included in more disease panels and in an expanding number of panels involved in the search of over 7,200 individuals. Overall, pathogenic small variants and intragenic deletions/duplications in these genes are rarely observed, but detectable by the available methods. Eight individuals were identified as having a pathogenic small variant in the *MAGEL2* gene representing the possible diagnosis of Schaaf–Yang syndrome if paternally defective (Fountain and Schaaf, 2016; Fountain et al., 2017; Patak et al., 2019).

## Discussion

The streamlined approach of NGS with MLPA has a strong theoretical foundation which is validated by real-life case analysis, as exemplified in our study. Compared to a serial approach using microarray and/or MLPA, our approach has an improved capability to directly and indirectly assess the underlying mechanism of Prader–Willi syndrome (PWS) and Angelman syndrome (AS). Furthermore, as this platform is based on MS-MLPA and whole-exome sequencing data obtained routinely in that order in commercial molecular genetic testing laboratories, it offers increased flexibility. From a clinical perspective, both PWS and AS often present with non-specific findings, particularly during infancy. For PWS, a combination of hyperphagia-based obesity that occurs after infancy, hypotonia, and intellectual disability is typically present, but can vary widely and is age-dependent. For AS, hypotonia and growth issues (e.g., obesity) can also be present, but usually not associated with hyperphagia, as well as more severe intellectual disability, epilepsy, ataxia, microcephaly, and other clinical features (Butler and Duis, 2020). As these symptoms are similar to those seen in individuals with Rett syndrome and other similarly related genetic conditions, a specific diagnosis is typically not made until additional molecular tests or the obtained results are available, requiring more time, resources, and effort. Rett and other related gene disorders, CNV, and point mutations of other causative genes would also be detected utilizing the WES data with this streamlined molecular diagnostics approach, specifically for Prader–Willi and Angelman syndromes. In addition, chromosome 15 pathogenic recessive variants in PSW or AS males or females when present with segmental or total isodisomy 15 UPD subclasses, or in skewed X-inactivation in females with any UPD subclass, could impact the clinical phenotype.

Our previous experience with testing of thousands of patients presenting for genetic laboratory services using both exome sequencing with bioinformatics and MLPA shows the rarity of copy number variation or AOH of probes within the 15q11-q13 region (0 to < 1%, respectively), further generating evidence for informative use of these combined methods in screening for *SNRPN* and related genes in this region. Hence, in summary, we recommend gene panel testing using a whole exome backbone combined with copy number analysis, AOH analysis, and methylation-specific MLPA, as described for individuals suspected of potentially having PWS or AS with utility and accuracy demonstrated in our study. This streamlined approach for molecular diagnostics is anticipated to be as accurate as or higher than the approximate 99% rate for PWS utilizing methylation PCR analysis alone, with a single *SNRPN* probe. However, methylation-sensitive MLPA assays multiple methylation probes representing two separate imprinted genes (*SNRPN* and *MAGEL2*) for PWS. In addition, methylation PCR does not have the capability of identifying the specific molecular genetic class or defect.

The streamlined approach we describe would identify the molecular genetic class and subtypes, as illustrated, and would be informative in 97% of patients with PWS and presumably in AS, as well. As this approach would identify point mutations and copy number changes [or imprinting center (IC) microdeletions], if present in those with a non-deletion status [i.e., maternal heterodisomy 15 vs. imprinting center defect (epimutation)], additional genotyping using chromosome 15 markers and parental DNA would not be required as the microdeletion form of the IC defects would have been ruled out, hence a low recurrence risk for subsequently affected children. If an IC microdeletion was found, the recurrence risk may be as high as 50% (Butler, 2016). This streamlined method will be further tested in a larger group of patients presenting with PWS and AS, and accumulated data will be helpful. This testing approach should also identify patients with mosaicism, a potential subgroup of patients who are currently underreported. Several genetic syndromes could similarly be tested for CNV, uniparental disomy, methylation status, and gene variants. Those include chromosome disorders such as 15q duplications, microdeletion syndromes with differing deletion sizes, and sequencing of syndromic candidate genes (e.g., Smith–Magenis, Williams, 22q11-q13, and 16p11-p13). Gene variants, uniparental disomy, and the methylation status of imprinting disorders such as Beckwith–Weidemann, Silver–Russell, GNAS-related defects, and Temple (e.g., Butler, 2020) could also be identified.

Our study shows the value of a streamlined molecular diagnostic approach to accumulate information required to rule out many other conditions with similar neurodevelopmental–functional findings having gene variants or copy number changes. These diagnoses are important for disease surveillance, treatment, and accurate genetic counseling and testing of at-risk family members. The authors would encourage the use of this methodology for confirming or establishing the diagnosis of individuals with these two genomic imprinting disorders. The addition of analysis of chromosome 14 and maternal disomy 14 may be warranted to assess for Temple syndrome, which can resemble PWS (Butler, 2020). For individuals negative for this streamlined testing with phenotypes similar to PWS with obesity, an expanded panel of genes can be considered to test diseases recognized as syndromic obesity syndromes, such as Alstrom, Bardet–Beidel, Cohen, Carpenter, Kabuki, WAGR, or Fragile X, or monogenic causes (e.g., *LEP*, *LEPR*, *BDNF*, *FTO*, *SH2B1*, *POMC*, *MCR4*, *TUB*, *AGRP*, *UCP1*, *CART*, *NEGR1*, and *PPARG*) (e.g., Bell et al., 2005; Choquet and Meyre, 2010; Butler, 2016; Kaur et al., 2017). Alternatives to this approach could include reflex testing using MLPA alone initially to identify individuals who are positive for the most common PWS/AS events and follow-up exome if negative or if further delineation of the mechanism is needed, as represented in our laboratory testing flowchart (Figure 1).

## Data Availability Statement

The original contributions presented in the study are publicly available. This data can be found here: https://www.ncbi.nlm.nih.gov/bioproject/687521.

## Ethics Statement

The studies involving human participants were reviewed and approved by the IRB, Kansas University Medical Center. The patients/participants provided their written informed consent to participate in this study.

## Author Contributions

SS and MGB designed the study and wrote the original manuscript. SS, MG, ML, JF, WS, H-YY, MT, JL, and HG participated in the laboratory and bioinformatics activities with generation of laboratory results and interpretations. MGB and WH provided the samples. All authors contributed to the writing, reviewing, and commenting and agreed to the final version of the manuscript prior to submitting for publication.

## Conflict of Interest

SS, MG, ML, JF, WS, H-YY, MT, JL, and HG were employees of Fulgent Genetics, a for-profit firm offering genetic testing as a fee for service. The remaining authors declare that the research was conducted in the absence of any commercial or financial relationships that could be construed as a potential conflict of interest.
